# Renin–Angiotensin–Aldosterone System and Immunomodulation: A State-of-the-Art Review

**DOI:** 10.3390/cells10071767

**Published:** 2021-07-13

**Authors:** Driss Laghlam, Mathieu Jozwiak, Lee S. Nguyen

**Affiliations:** Research and Innovation of CMC Ambroise Paré (RICAP), CMC Ambroise Paré, 25-27 Boulevard Victor Hugo, 92200 Neuilly-sur-Seine, France; driss_laghlam@hotmail.com (D.L.); mathieu.jozwiak@aphp.fr (M.J.)

**Keywords:** renin–angiotensin system, oncology, shock, transplantation, immunomodulation

## Abstract

The renin–angiotensin system (RAS) has long been described in the field of cardiovascular physiology as the main player in blood pressure homeostasis. However, other effects have since been described, and include proliferation, fibrosis, and inflammation. To illustrate the immunomodulatory properties of the RAS, we chose three distinct fields in which RAS may play a critical role and be the subject of specific treatments. In oncology, RAS hyperactivation has been associated with tumor migration, survival, cell proliferation, and angiogenesis; preliminary data showed promise of the benefit of RAS blockers in patients treated for certain types of cancer. In intensive care medicine, vasoplegic shock has been associated with severe macro- and microcirculatory imbalance. A relative insufficiency in angiotensin II (AngII) was associated to lethal outcomes and synthetic AngII has been suggested as a specific treatment in these cases. Finally, in solid organ transplantation, both AngI and AngII have been associated with increased rejection events, with a regional specificity in the RAS activity. These elements emphasize the complexity of the direct and indirect interactions of RAS with immunomodulatory pathways and warrant further research in the field.

## 1. Introduction

The renin–angiotensin system (RAS) has long been considered the pinnacle of homeostasis in cardiovascular physiology. Its main function involves regulation of blood pressure, via direct and indirect means, through electrolyte balance, and trophic and vasomotor functions. While historically associated with these basic functions, the role of inflammation in cardiovascular diseases has been more and more described, and a critical role of RAS in inflammation regulation has been suggested.

**Figure 1:**
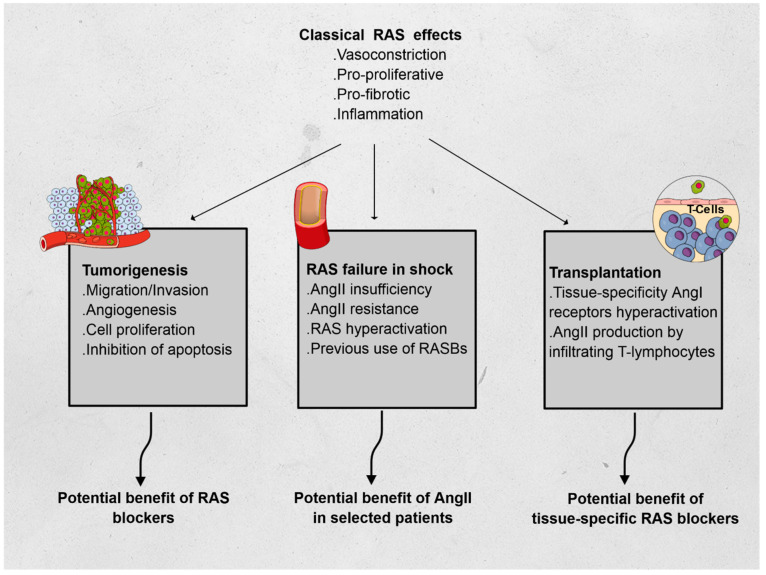
Effects of the renin–angiotensin system (RAS) and its features in oncology, vasoplegic shock, and transplantation. Abbreviations: AngI: angiotensin I, AngII: angiotensin II.

Using three different fields of application, we hereafter describe non-classical RAS functions, relying on the immunomodulatory features of RAS: oncology, intensive care, and transplantation (see Figure 1).

## 2. Part 1: Renin–Angiotensin System Blockers and Malignancies

Renin–angiotensin system blockers (RASBs), which include angiotensin-converting enzyme inhibitors (ACEIs) and angiotensin-2 receptor 1 blockers (ARBs), have been used as antihypertensive drugs for several decades, showing benefits in terms of morbidity and mortality in cardiovascular diseases.

Only recently have other organ-protective effects been described. These encompassed inhibition of cardiac hypertrophy, remodeling, fibrosis, and reactive oxygen species (ROS) production. Notably, numerous observations of RASB treatments in patients with solid cancers showed some benefits, even towards survival that was free of cancer evolution. In the following section, we describe the interactions between RAS and cancer.

### 2.1. Pathways Related to RAS

Angiotensinogen (synthesized in the liver) is the only precursor protein of RAS and the source of all angiotensin peptides. Angiotensinogen is primarily cleaved in blood circulation by renin (protease synthesized in the kidney), to angiotensin I (AngI), which in turn is processed by angiotensin converting enzyme (ACE) (secreted in lung) to produce the active octapeptide, angiotensin II (AngII). AngII actions are primary mediated through the binding of its two receptors, type 1 (AT1R) and type 2 (AT2R), which belong to the G protein-coupled receptor superfamily. The important cardiovascular actions of AngII, including regulation of arterial blood pressure with short-term vasoconstriction, aldosterone release, and water and salt balance, are predominantly mediated by AT1R in target tissues, such as blood vessels, kidney, brain, and heart [1].

**Figure 2:**
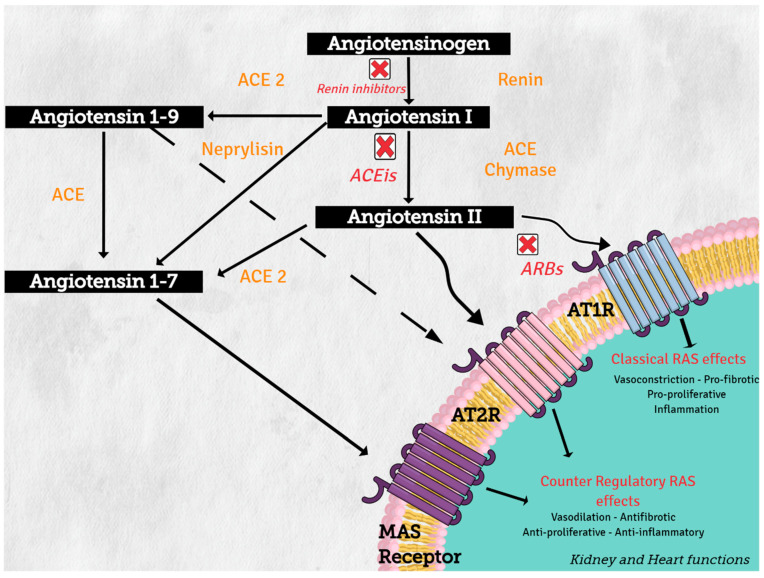
Renin–angiotensin system: classical and counter regulatory pathways. Abbreviations: ACE: angiotensin converting enzyme; ACE 2: angiotensin-converting enzyme type 2, ACEIs: angiotensin-converting enzyme inhibitors; ARBs: angiotensin-2 receptor 1 blockers, AT1R: angiotensin II receptor type1, AT2R: angiotensin II receptor type 2.

On the other hand, increasing evidence supports the concept of a counter regulatory renin–angiotensin system with opposite effects in cardiovascular physiology and disease [2]. Angiotensin converting enzyme 1 (ACE2) and neprilysin (NEP) are part of this counter regulatory system. ACE2 and NEP can cleave AngII to produce angiotensin 1–7 (Ang 1–7), and ACE2 cleaves AngI to generate angiotensin 1–9 (Ang 1–9) (see Figure 2). These two peptides mediate the antagonistic effects of AngII. Angiotensin 1–7 binds to the proto-oncogen Mas receptor, which leads to vasodilation, antihypertensive, and antifibrosis effects [3], while Ang 1–9 can activate AT2R to trigger natriuresis and NO production, thus mediating vasodilatory effects and reducing blood pressure [4].

Over the past decade, growing evidence has shown that an altered immune system is likely to be a key contributor to the development of hypertension. Consistent evidence demonstrates that various immune cell subsets infiltrate blood vessels, kidneys, heart, and/or the autonomic nervous system during hypertension, and that the targeted depletion of specific immune cell subsets or inhibition of their functions protects against hypertension in animal models. However, no definitive mechanistic explanation has been established for how immune cells influence organ functions to promote increases in blood pressure [5]. Pro-inflammatory T cell-derived cytokines, such as IFN-γ and TNF-α (from CD8+ and CD4 + Th1) and IL-17A (from the γδ-T cell and CD4 + Th17), were found to exacerbate hypertensive responses mediating both endothelial dysfunction and cardiac, renal, and neurodegenerative injury. B cell activation and production of autoantibodies (anti-Hsp70, anti-Hsp65, anti-Hsp60, anti-AT1R, anti-α1AR, and anti-β1AR) were also associated with hypertension, while hypertensive responses can be inhibited by T regulatory lymphocytes (Tregs) and the anti-inflammatory IL-10 [6]. Other elements have been associated with a modulation of response of lymphocytes T to AngII, most lately sympathetic tone [7], as suggested by stress-related hypertension [8].

The role of antihypertensive drugs in modulating angiotensin-converting enzyme in immune function and inflammation could be genetically related. A recent study gave indications of the beneficial effects of ACEIs on immune function and inflammation, using a Mendelian randomization in the largest available genome-wide association studies, genetically predicted the effects of ACEI’s (single nucleotide polymorphisms rs4968783 and rs4291 in ACE) increased lymphocyte percentage (0.78, 95% confidence interval (CI) 0.35, 1.22) and decreased neutrophil percentage (−0.64, 95% CI −1.09, −0.20), and possibly lowered TNF-α (−4.92, 95% CI −8.50, −1.33). This effect of ACEIs is not due to, or at least is not totally due to ACE 2 expression, because, in contrast ARBs, which also affect ACE2 expression, they were not found in this study to affect lymphocyte, neutrophil, or TNF-α [9].

Qin et al. demonstrated that AngII induces the expression of proteasome protein subunits and predominantly stimulates Th1 differentiation. This effect of AngII in proteasome activities was associated with the activation of the AT1R/PKA signaling pathway, which in turn promotes the degradation of MAKP-1 (mitogen-activated protein kinase) and IκBα and the activation of the STAT1 and NF-κB pathways, which eventually lead to Th1 cell differentiation. In addition, inhibition of proteasome activation or AT1R markedly attenuated these effects [10].

The activated AT1R coupled with a classical Gq/11 protein can also activate multiple intracellular signal transduction pathways in cardiovascular, renal, and immune cells; with several tyrosine kinases, including receptor tyrosine kinases (epidermal growth factor receptor (EGFR), PDGF, and insulin receptor) and nonreceptor tyrosine kinases (c-Src family kinases, Ca2-dependent proline-rich tyrosine kinase 2 (Pyk2), focal adhesion kinase (FAK), and Janus kinases (JAK)). AT1R also activates serine/threonine kinases, such as PKC and MAPK (extracellular regulated kinases (ERK) 1/2, p38MAPK and Jun N-terminal kinase). Transcription of nuclear factor κB (NFκB) participates in the Raf pathway and generates ROS via NAD(P)H oxidase activation (in cardiovascular cells, leukocytes, and monocytes) [11,12,13]. This link with tyrosine kinase-related pathways and their downstream effectors explains the well-established role of RAS in the growth and remodeling of the vasculature, kidney, and heart, which occurs during metabolic disturbances and cardiovascular disease [13,14]. Although AT1R mediates most of the known vasoactive effects of AngII, AT2R via the activation of phosphatases, GMPc, and phospholipase-A2, and mainly by antagonizing AT1Rs, contribute to the regulation of blood pressure and renal function and decrease cell proliferation in the kidney glomerular capillary wall while increasing apoptosis and cell differentiation [15].

### 2.2. RAS, Inflammation, Diabetes, and Metabolic Syndrome

It must be emphasized that the association between RAS and inflammation has also been observed in the context of metabolic syndrome, including diabetes, mostly through ACE2 activity. Indeed, ACE2 is expressed in liver, skeletal muscles, and adipose tissue, and any disturbance to ACE2/Ang-(1–7) activity could lead to a glucose homeostasis disorder. Moreover, loss of the ACE2 gene in mice leads to hepatic fibrosis and impaired glucose homeostasis through an elevated hepatic reactive oxygen species level, an increased oxidative stress, and inflammation in the liver, leading to an impairment in insulin signaling [16]. In adipose tissue, ACE2 deficiency worsens inflammation in response to diet-induced obesity in mice [17]. Incidentally, RAS activity in T-cells was also found to be disturbed in obese patients, in whom AngII stimulation was observed [18]. Conversely, overexpression of ACE2 or Ang-(1–7) administration improves these metabolic disorders, i.e., glycemic control and insulin sensitivity [19,20,21]. Indeed, mechanistically, Ang-(1–7) rescues the insulin signaling pathway by stimulating PKB phosphorylation, a main mediator of the insulin signaling pathway, which will then activate the downstream glycogen synthase kinase-3β in the liver and skeletal muscles, resulting in a decrease in glycemia through glycogen storage [22] in several murine models of diet-induced insulin resistance, such as high-fat diet fed mice or in fructose-fed rats [23,24]. In adipose tissue, activation of ACE2/Ang-(1–7) prevents inflammation and oxidative stress induced by a high-fat diet and increases glucose uptake and adiponectin level [25,26,27], while its disturbance results in a lower insulin-dependent glucose uptake and adiponectin secretion [28].

In addition to the effects of the inhibition of the above-mentioned alternate effects of RAS, in the context of metabolic diseases, such as obesity, T2DM, or nonalcoholic fatty liver disease, plasmatic Ang II is positively correlated with body weight and is associated with insulin resistance, suggesting that ACE/Ang II activity is upregulated in those metabolic disorders [29]. In addition, on a tissue scale, Ang II was associated with increased insulin resistance through oxidative stress, leading to hepatic fibrosis and cirrhosis, provoking an impairment of insulin signaling [30]. In skeletal muscles, Ang II also induces a decreased glucose uptake and impairs insulin sensitivity [31], while in adipose tissue, it inhibits adiponectin secretion and insulin signaling through an increased oxidative stress [32]. These elements emphasize the pro-diabetogenic effects of classical RAS effects, in parallel to those of inflammation.

### 2.3. RAS and Tumorigenesis Pathways

**Figure 3:**
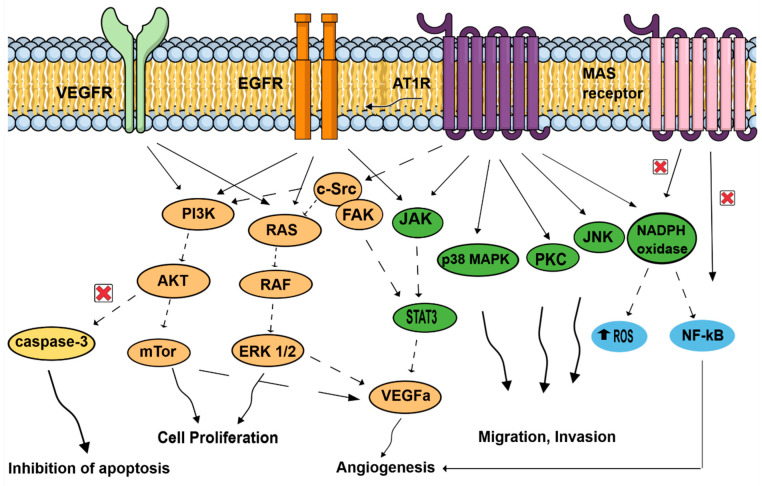
Signal transduction pathways induced by the renin–angiotensin system, associated with cell proliferation, migration, invasion, inhibition of apoptosis, and angiogenesis. AT1R: angiotensin II receptor type 1; AKT: protein kinase B; c-SRC: non-receptor tyrosine kinase c-SRC protein; EGFR: epidermal growth factor receptor, JAK: Jun activating kinase; JNK: Jun N-terminal kinase; MAPK: mitogen-activated protein kinase; mTor: mechanistic target of rapamycin; NADPH oxidase: nicotinamide adenine dinucleotide phosphate oxidase; NFκB: nuclear factor κB; PI3k: phosphoinositide 3-kinase; ROS: reactive oxygen species; STAT3: signal transducer and activator of transcription 3; VEGFA: vascular endothelial growth factor A; VEGFR: vascular endothelial growth factor receptor.

The effects of activating the RAS pathway axis signaling has been mostly studied in tumor cells. On top of the usual blood pressure regulation role of ATR1, more recent observations showed that AT1R mediates several pathological events associated with activated RAS, such as upregulation of cell proliferation, inhibition of apoptosis, motility, migration, invasion, and angiogenesis (see Figure 3) [33,34,35,36]. In contrast, the Ang (1,7)-MAS receptor and the Ang II-AT2R pathways are thought to antagonize many of the cellular actions of the Ang II-AT1R axis. Tumors cells, but also important components of the tumor microenvironment, such as endothelial cells and fibroblasts, can generate and express RAS components promoting angiogenesis [37]. Neutrophils and macrophages are also capable of and use these RAS signaling pathways to produce and secrete growth factors (VEGF), cytokines (IL-1, IL-6, TNFα), and generate reactive oxygen species in hypoxic and inflammatory environments [38]. As tumorigenesis involves angiogenesis, the involvement of ATR1 may be easily fathomed as a complex phenomenon involving several signaling pathways in endothelial cells: EGFR; MAPK, and Erk1/2 through AngII activating collagen I gene [39,40] and the transcription of growth-related factors [41,42,43]. Likewise, vascular endothelial growth factor A (VEGF-A) expression in endothelial cells, essential to neovascularization within the tumor and for tumor growth, is triggered by Ang-II through AT1R and ERK1/2 signaling and, thus, promotes neoangiogenesis in various tumors (pancreatic, ovarian, hepatocellular, bladder cancers) [44,45,46,47].

On top of promoting angiogenesis, AngII/AT1R might couple to malignancy through the transactivation of EGFR, thereby hijacking downstream signaling pathways that were linked to malignant transformation, and favors cell proliferation of cancer cells by activating molecular cascades: PI3K/AKT pathway (breast cancer) [48], paired homeobox 2 (PAX2), STAT3 (signal transducer and activator of transcription 3) and JAK II (Jun activating kinase) pathways in prostate tumors [49], and RAS/RAF/ERK1/2 pathways. AngII/AT1R activation favors invasion and survival via the NF-κB pathway (breast and gastric tumors) [48,50], which is, in contrast, inhibited by Ang (1,7)-MAS receptor. Finally, activation of ATR1 plays an antiapoptotic role by increasing cell survival [50], and by suppressing the activity of caspase-3 via the activation of the PI3K/Akt pathway in vascular endothelial cells [51].

These observations illustrate the role of RAS in tumorigenesis and suggest a potential synergistic role of RASBs in cancer treatment: tumor progression may be slowed down through inhibition of proliferation and neovascularization, promotion of tumor cell apoptosis, and enhancement of anti-cancer drug delivery [52].

The effects of activating Ang II/AT1R, Ang II/AT2R (not represented here), and Ang 1–7/MAS receptor axis signaling has been mainly studied in tumor cells. In contrast to the Ang II/AT1R that mediates several pathological events associated with activated RAS, such as upregulation of cell proliferation, inhibition of apoptosis, motility, migration, invasion, and angiogenesis, the Ang (1,7)-MAS receptor and Ang II-AT2R (not represented here) pathways are thought to antagonize many of the cellular actions of the Ang II-AT1R axis. In cancer cells, activated AT1R subunits lead to activation of signaling cascades, including the JAK/STAT3, cSRC/FAK, PKC and JNK pathways, promoting migration and invasion. AT1R might couple to malignancy through the transactivation of EGFR, thereby hijacking downstream signaling pathways that were linked to malignant transformation. EGFR pathways are related to JAK/STAT3, RAS/RAF/ERK1/2, and PI3K/AKT/mTor pathways, increasing cell proliferation. On the contrary, NF-kB, which promotes invasion and survival, is inhibited by Ang (1,7)-MAS receptor activation. Important components of the tumor microenvironment, such as macrophages, endothelial cells, and fibroblasts, can generate and express RAS components. Endothelial cells use AT1R, but also MAS receptor pathways. Activated AT1R leads to MAPK pathways, favoring mobility. VEGF receptors in endothelial cells conducted to p38MAPK, FAK, and to PI3K/AKT/capsase3 pathways, leading to inhibition of apoptosis and proliferation. In addition, NADPH oxidase and NF-kB promote angiogenesis in endothelial cells. In fibroblasts, activated AT1R is associated with MAPK and ERk1/2 pathways. Neutrophils and macrophages are also capable of generating and expressing RAS component, and use these signaling pathways to produce and secrete growth factors (VEGF) and cytokines (IL-1, IL-6, TNFα; not represented here). In hypoxic and inflammatory environments, neutrophils and macrophages, in response to AngII stimulation, generate reactive oxygen species, contributing to proliferation and angiogenesis.

### 2.4. Impact of RASBs Use in Cancer

The effects of RASBs in cancer, drawn from observational studies, remain elusive, yielding conflicting results. Furthermore, RASB meta-analyses and retrospective studies may not be reliable because of inherent bias as they were never designed to explore any pro- or antitumoral effects.

In 1998, Lever et al. observed, in a retrospective cohort study based on 5207 patients in Scotland, that the relative risks (RR) of incident and fatal cancer among 1559 patients receiving ACEIs were 0.72 (95% CI 0.55–0.92) and 0.65 (0.44–0.93), compared to patients not treated using ACEIs [53]. Meanwhile, another observational study in Denmark reported no protective effect. Cancer incidence among 17,897 patients treated using RASBs was analyzed, with an expected incidence based on county specific cancer rates during an 8-year study period, and the standardized incidence ratios was 1.07 (95% CI, 1.01–1.15) [54].

Thereafter, observational studies surrounding RASBs use and overall cancer still yielded conflicting results. A summary of the main meta-analyses of RABs use and overall risk of cancer occurrence is shown in Table 1. As an example, while one meta-analysis of randomized studies showed an increased risk of new cancer occurrence (RR 1.08, 95% CI 1.01–1.15; *p* = 0.016) [55], another found lower incidence of cancer in observational studies (RR 0.82, 95% CI 0.73–0.93), but not in the randomized controlled trials (RR 1.00, 95% CI 0.92–1.08) [56]. Another observational study did not find a significant association between RASBs and the overall risk of cancer (RR = 0.96 (0.90–1.03)) [57].

Studies surrounding RASBs use and specific organ cancer risk are presented in Table 2. When focusing on specific cancers, RASBs have been associated with a decreased risk of colorectal [58,59], keratinocyte [60], and prostate cancer [61]. In other cancers, association was null, particularly in liver [62] and breast cancer, where conflicting results coexist [63,64]. Finally, in a recent meta-analysis, based on 31 observational studies, RASBs were associated with a risk increase of bladder and kidney cancer [65].

Finally, duration of RASBs treatment has also been suggested as a relevant element: long-term use of ACEIs was associated with an increased risk of lung cancer through the accumulation of bradykinin [66] and substance P [67]. ACEI use seems to lead specifically to an increased risk of lung cancer compared to ARBs. This association was reported in a large population-based cohort in the UK [68], later replicated in an Asian cohort [69].

### 2.5. RASBs and Survival with Cancer

As previously described, RASBs could be a candidate as an adjunctive cancer therapy using the link between RAS and the tumorigenesis pathways, inhibition of neovascularization, and prevention of cancer-treatment-related adverse events. A summary of the main RABs and cancer survival studies is shown in Table 3.

First, on top of inhibition of tumor growth and recurrence, RASB use may also mitigate cancer-treatment-related adverse events, which worsen the prognosis of these patients. This protective association is well established in prevention of cardiotoxicity of anticancer drug treatments, in solid cancers and hemopathy [70]. Breast cancer studies showed a great deal of evidence of the beneficial effects of RASBs after treatment using anthracyclines and immunotherapy, including trastuzamab (see Table 4). In three randomized-controlled studies, use of RABs during anthracyclines and trastuzumab regimens was found to reduces cardiotoxicity (treatment-mediated decline in LVEF was attenuated). Moreover, a large retrospective study found a reduction in cardiotoxicity (HR 0.77, 95% CI 0.62–0.95) and of all-cause mortality (HR 0.79, 95% CI 0.70–0.90) in RAB users compared with the nonexposed group. Interestingly, there was a dose-dependent interaction as starting RAB therapy ≤6 months after the initiation of trastuzumab/anthracyclines and having an exposed duration ≥6 months were also associated with decreased risk of cardiotoxicity and all-cause mortality [71,72,73,74,75]. In addition, prevention of radiation injury and arterial hypertension induced by anti-VEGF therapies was also suggested, although the degree of evidence is less obvious [76].

Second, RAS blockade was associated with improved outcomes in several cancer types. Indeed, even though patients treated using RASBs were more likely to present several cardiovascular comorbidities on top of hypertension, and hence a greater theoretical risk of non-cancer mortality, improved survival was still observed in several cohort studies.

Few prospective studies exist, and only with a limited number of patients, yet they support that RASB use versus placebo, in addition to standard cancer treatment, may be associated with a reduction in prostate cancer recurrence after radical prostatectomy [77], and in hepatocellular carcinoma [78].

Most evidence of survival improvements in RASB users come from retrospective studies of various cancer types, at different stages. Two recent meta-analyses showed that the use of RASBs combined with chemotherapy can lead to a significant reduction in the risk of cancer recurrence and mortality [79,80].

RASBs have been associated with significant improvements to complete response in association with neoadjuvant radiation [81], and with less recurrence of left-sided and early stage colorectal cancer [82]; while a meta-analysis found an increased survival with system digestive malignancies [83]. In a large nationwide Finnish cohort, the authors found a significant reduction in mortality due to breast cancer in RASB users, also featuring a dose-effect, suggesting a mechanistic association [84]. Similarly, concurring elements supported that urological cancer survival improved with RASB use [85,86,87,88,89]. In non-small-cell lung cancer, RASB use with platinum and taxol chemotherapy was associated with improved survival [90,91]. However, ACEI use was found to be deleterious in association with anti-PD-1 (pembrolizumab, nivolumab) and anti-PD-L1 (durvalumab) immune checkpoint blockers in advanced non-small-cell lung cancer. Authors found that patients taking concomitant ACEIs had an immunosuppressed state, suggested by less M1 macrophages, activated mast cells, natural killer (NK) cells, and memory activated T cells [92].

Despite accumulating evidence, prospective studies are still warranted to prove a potential beneficiary effect of RASB as an adjuvant therapy in cancer.

## 3. Part 2: Renin–Angiotensin System in Intensive Care Medicine

### 3.1. RAS in Vasodilatory Shock

Sepsis is the main cause of vasodilatory shock. This shock state combines both macrocirculatory disorders with the association of hypovolemia, peripheral vasodilation, cardiac dysfunction, and microcirculatory disorders with microcirculation impairment. Macrocirculatory disorders induce an imbalance between oxygen supply and delivery, leading to inadequate tissue perfusion and cellular hypoxia, whereas microcirculatory disorders induce an impairment of peripheral oxygen extraction and, thus, of tissue oxygenation [93].

In response to shock, several physiologic and adaptive mechanisms are triggered to restore arterial pressure [94]. Among them, the activation of RAS [95,96,97,98,99,100,101,102,103,104], with an increase in renin secretion by the juxtaglomerular cells, resulting in elevated AngII plasma levels, vasoconstriction, aldosterone synthesis from the adrenal cortex, and vasopressin release [93,94,105].

#### 3.1.1. RAS Activation in Sepsis

RAS activation in vasodilatory shock has been mainly demonstrated in experimental studies [101,106,107,108,109,110,111,112,113]. The vasopressor activity of AngII allows vascular tone and restoration of arterial pressure through both venous and arterial constriction [114], as well as regional blood flow regulation and, especially, kidney blood flow regulation [115,116]. In addition to its hemodynamic effects, RAS, and especially AngII, modulate several biological pathways, including inflammation and cell growth [117], coagulation, and mitochondrial function [117,118].

Although RAS activation is a physiologic and adaptive response to vasodilatory shock, excessive RAS activation could be deleterious. First, excessive AngII production is associated with marked vasoconstriction [103], risking mesenteric ischemia and microvascular thrombosis [96,99,104,119,120,121]; moreover, excessive AngII production impairs mitochondrial function [122,123,124,125], stimulates mitochondrial reactive oxygen species production in endothelial cells [126], and, thus, results in oxidative stress and endothelial injury [109,127,128]. It has been suggested that both oxidative stress and endothelial injury may contribute to the development of organ failure, such as acute respiratory distress syndrome [108,113,115] or acute renal failure [102,129].

#### 3.1.2. RAS Failure in Sepsis

In sepsis, experimental studies showed that receptors of AngI and AngII [101,107,130,131,132], as well as different intracellular pathways involved in the regulation of vasodilatory mediators synthesis [107,132], were down-regulated or less sensible to AngII stimulation. Hypothesized mechanisms include excessive nitric oxide synthesis [99,101,107] and the activation of specific ATP-sensitive potassium channels located in the membrane of vascular smooth cells [133]. In addition, sepsis-induced endothelial injury has been associated with ACE deficiency, preventing the conversion of AngI to AngII [134,135], contributing to a relative decrease in AngII plasma levels, which was observed in patients with septic shock [128].

Taken together, this relative decrease in AngII plasma levels, combined with decrease in sensitivity to AngII stimulation, may result in a relative lack of endogenous catecholamines, since AngII naturally induces catecholamine secretion by the adrenal cortex [136]. In the most severe cases, death may occur due to refractory vasodilatory shock with multiple organ failure [99,115,137].

### 3.2. RAS to Treat Vasodilatory Shock

Because vasodilatory shock is primarily characterized by vasodilation, vasopressor therapy is the mainstay of symptomatic treatment. Numerous vasopressors are available, each with its own side effects, and each acting on veins and arteries through different pathways after activation of specific receptors [93]. While some vasopressors are natural hormones that exert a vasoconstrictive effect through receptor activation (norepinephrine, epinephrine, vasopressin, AngII), most recent vasopressors, such as selepressin, are modifications of natural hormones. In patients with septic shock, the choice of vasopressor should take into account the fact that endotoxins block the ability of vascular smooth muscle to respond to vasoactive agents. In this regard, norepinephrine is currently the first-line vasopressor therapy in vasodilatory shock [93,138,139], followed by epinephrine and vasopressin. However, given the potential deleterious effect of high-dose norepinephrine administration [128,140], combined with the importance of RAS activation as an adaptive mechanism, and the potential deleterious impact of excessive RAS activation in vasodilatory shock, there has been a growing interest in investigating the effects of AngII and RASBs as treatments in vasodilatory shock.

#### 3.2.1. AngII Use in Sepsis

Historically, the first studies in patients with shock, showing that AngII had similar effects to those of norepinephrine on arterial pressure, started in the 1960s [141,142]. Since then, experimental studies have shown that the administration of AngII in animal septic models allowed improvements to arterial pressure and renal function [110,111,118]. Small clinical studies also suggested potential interest in AngII administration for patients with vasodilatory shock [143,144,145], or with refractory septic shock, unresponsive to high-dose of norepinephrine [137,146]. More recently, a single-center pilot study, including 20 patients with septic shock requiring multiple vasopressors, showed that the administration of AngII allowed restoring mean arterial pressure with a catecholamine-sparing effect and without significant adverse renal effects, despite the marked vasopressor activity of AngII on the renal vasculature [130]. Of note, in this study, the initial AngII dosage, which was deemed appropriate, ranged from 2 to 10 ng/kg/min [130].

In 2017, a multicentric randomized double-blind, placebo-controlled trial (ATHOS-3) was conducted to confirm the potential use of AngII in patients with high-output catecholamine-resistant vasodilatory shock [131]. It included 334 patients with persistent vasodilatory shock, despite adequate fluid resuscitation, and administration of high doses of norepinephrine for a minimum of 6 h and a maximum of 48 h. Compared to placebo, AngII allowed to achieve a predefined mean arterial pressure target, along with a decrease in catecholamine dosage. However, mortality rates were similar in both groups of patients [131]. After post hoc analyses, those who benefited most from AngII administration were those with the most severe presentations with a relative AngII deficiency [147] and markedly elevated serum renin concentrations at baseline (ref. AJRCCM 2020). In a second post hoc analysis, in a subgroup of 105 patients with acute kidney injury requiring renal replacement therapy, AngII was associated with a lower 28-day mortality rate, a better correction of hypotension, and a faster recovery of kidney function [148]. In addition to its effects on arterial pressure and kidney function, AngII administration might also reduce fluid balance in patients with vasodilatory shock through aldosterone synthesis, involved in water and salt balance, and, thus, in volume regulation. Despite the fact that no study has evaluated the effect of AngII administration on patient fluid balance so far, this potential beneficial effect of AngII must be kept in mind, since a positive fluid balance in patients with septic shock is independently associated with mortality [149,150,151].

Despite these encouraging results, the safety of AngII is still matter of debate, in terms of its marked vasopressor activity [94,152]. In the ATHOS-3 trial, the proportion of serious adverse effects, such as ischemic events (digital, gut, myocardial) and cardiac arrhythmias, were similar in patients receiving AngII or placebo, and was around 90% [131]. A systematic review of 1124 studies, including 31,281 patients receiving AngII, concluded that AngII-induced side effects were infrequent with ≤300 reported side effects, and that the most common side effects were transient headaches, abnormal chest sensations, and orthostatic symptoms following discontinuation. Interestingly, only two deaths were causally related to AngII administration, none of which occurred in patients with vasodilatory shock [153]. Nevertheless, only 13 of the included studies were conducted in patients with vasodilatory shock, making the external validity of these results questionable in the case of critically-ill patients. More recently, a sensitivity analysis of the ATHOS-3 trial showed that, in 48% of patients who were included, AngioII dosage could be decreased from 20 ng/kg/min to ≤5 ng/kg/min within the 30-min period following treatment initiation [128]. Compared to patients receiving a higher AngII dosage, these patients had a better MAP response and a lower 28-day mortality rate. Interestingly, along with better outcomes, this subset of patients also experienced less serious side effects [128]. Altogether, these findings suggested that a low dose of AngII may be effective and safe for patients with vasodilatory shock, and may also be an early indicator of which patients are more likely to respond to AngII administration.

To summarize, AngII administration appears to be a promising and relatively safe therapy in the early resuscitation of patients with vasodilatory shock, especially in the more severe and hypotensive patients and/or in patients with acute kidney injury requiring renal replacement therapy. In the light of the results of the ATHOS-3 trial, the US Food and Drug Administration approved the use of AngII for the treatment of hypotension in adults with distributive shock [154].

#### 3.2.2. RASBs Use in Sepsis

Vasodilator therapy may also be interesting in patients with vasodilatory shock in terms of microcirculation, despite contradicting findings [155,156]. Given the potential deleterious effect of excessive RAS activation in terms of ischemic and inflammatory insults in patients with vasodilatory shock, RASBs might potentially have a place in the treatment of such patients, as demonstrated in preliminary experimental [120,121] and clinical studies [99]. In experimental models of septic shock, the administration of low-dose ARB improved survival rates, as well as renal and mesenteric perfusion, without any significant hemodynamic side effects [120,121]. Very recently, it was shown that, in septic models in rats, early administration of low-dose, of selective, ARB improved the vasoconstrictive response to AngII, whereas a high dose impaired the response to vasoconstrictors and worsened arterial hypotension, resulting in an increase in blood lactate levels and in renal failure [157].

In 40 surgical patients with sepsis, Boldt et al. demonstrated that the continuous infusion of an ACE inhibitor for 5 days decreased sepsis-induced endothelial dysfunction and risk of septic shock as compared to placebo; however, both treatment groups yielded similar survival rates [99].

Regardless of the type of RASB used, timing of administration may play an important role. Experimental [120] and clinical [99] data suggest that the earlier the administration of RASB, the better the survival and/or outcomes. A very recent retrospective cohort study including ≥50,000 patients hospitalized with sepsis confirmed that short-term mortality after sepsis was lower in patients who received RAS inhibitors when sepsis occurred [158]. In contrast, the administration of RAS inhibitors in established vasodilatory shock was associated with severe arterial hypotension and decreased organ perfusion [120,159]. Study design and retrospective analyses may preclude definitive conclusions, as the potential adverse effects of RASBs on organ perfusion pressure, including kidneys, in patients with impaired hemodynamics must be kept in mind before any administration.

### 3.3. The Future of RAS in Vasodilatory Shock

Despite encouraging preliminary results, the place of AngII in the therapeutic armamentarium for patients with vasodilatory shock still requires clarification. Choosing patients who will best benefit from AngII administration may be crucial. First, given the observed marked vasoplegia in patients with long-term treatment using RASBs, presenting with septic shock, AngII may be able to counterbalance a hypothetical ACEIs overdose [152]. In this regard, some studies showed that low AngII and ACE plasma levels [134] and low AngII state (defined by a high AngII/AngI ratio) [147] were accurate prognostic factors in patients with vasodilatory shock.

Second, only patients with a high cardiac output (and thus a preserved cardiac function) were included in the landmark ATHOS-3 trial [131]. Given that septic shock may be associated with myocardial dysfunction and myocardial injury [160], this would automatically preclude these patients from benefitting from AngII, as vasopressors in cardiogenic shock may be considered infra-physiological [161]. Finally, AngII administration could also be interesting for patients with vasodilatory shock and acute respiratory distress syndrome. Indeed, since ACE is located in pulmonary endothelium [162,163], these patients show decreased ability to convert AngI to AngII and may therefore show AngII deficiency, related to the severity of the acute respiratory distress syndrome [164].

## 4. Part 3: RAS and Transplantation

Allograft transplantation is associated with chronic activation of innate and adaptative immunity in recipient patients, leading to a chronic state of inflammation. The effects of RAS in solid organ transplant recipients remain debated. As previously described, RAS is associated with hypertension and its inherent cardiovascular effects. As hypertension and atherosclerosis are one of the main complications of long-term use of immunosuppressant drugs, the fact that hypertension can be treated may seem to be an obvious choice.

However, the effects of RAS on solid organ transplants, in exerting an immunomodulatory effect, are the focus of this section. As an illustration, we will cover heart and kidney transplantations.

### 4.1. RAS and Immunomodulation

#### 4.1.1. Tissue Specificity of AngI

General use of RASB in patients with hypertension showed that they, not only benefitted from a lower blood pressure, but also showed improvements in terms of kidney inflammation, independent from blood pressure, as attested by comparing ACEIs or ARB to calcium inhibitors in lupus nephritis [165], or decreasing inflammation in chronic kidney disease using RASB [166]. Moreover, murine models pinpointed the prominent role of AngI, as the main cause of immune-mediated fibrotic and inflammatory effects in target organs [167]. The role of increased AngI receptors activation in target organs was also highlighted in another murine study, highlighting the need to distinguish local tissue and systemic levels of RAS components.

Indeed, tissue-specific regulation of RAS components profoundly altered the mechanistic comprehension of hypertension when considering parenchymal renal cells [168]; similar for adaptative and innate immune response, organs may be considered equally dependent on local tissue specificity regarding sensitivity to RAS stimulation [169].

#### 4.1.2. AngII, a Cytokine Which Mediates Infiltration and Immune Activation

In solid organ transplantation, immune response is primarily targeted, but a non-targeted response is also involved [170]. By analogy, with non-immune renal diseases, mononuclear activation and infiltration of the interstitium of an organ may be seen as the manifestation of chemoattractant overexpression [171,172]. Moreover, an association between RAS overactivation and, conversely, blockade was observed with chemokines and adhesion molecules (MCP-1, osteopontine). In models of salt-sensitive hypertension, AngII-producing T-lymphocytes were observed in tubular interstitium, and oxidative stress, as well as hypertension, were reversed by the immunosuppressant drug, mycophenolate mofetil [173]. The response to neoantigens remains a hypothesis, as AngII has been associated with the promotion of antigen-presenting-cells [117].

#### 4.1.3. Other RAS Components and Immunity

In vitro, ACE was identified as a factor involved in the differentiation of endothelial, myeloid, erythroid, and lymphoid cell lineages from hemangioblasts derived from human pluripotent stem cells [174]. Moreover, ACE was associated with carboxypeptidase activity, cleaving major histocompatibility complex I peptides in antigen-presenting cells, thus allowing to alter the immune repertoire [175].

ACE2 and Ang 1–7 have both been associated with anti-inflammatory effects. ACE2, in particular, was associated with a decrease in the proinflammatory cytokines secreted by macrophages, including TNF-α and IL-6 [176]. Consequently, the accumulation of Ang 1–7 through catabolism in AngII may also be invoked when describing the anti-inflammatory effects of ACE2; the inherent anti-inflammatory effect of Ang 1–7 was shown in murine models of cardiac injury with diabetes and hypertension [177]. Interestingly, the effects of Ang 1–7, through a specific receptor, have been hypothesized in regards to persisting effects in Mas-receptor-deficient rodents [178].

AngI receptors have been shown to have distinctive effects, when considered for myeloid and lymphoid cells rather than renal and vasculature cells. Indeed, while AngI receptor activation in renal or vasculature cells is associated with an overall pro-inflammatory effect, the opposite was observed when activated in myeloid and lymphoid cells in murine models [179].

Several immune cell lineages, including T and NK cells, showed functional renin and ACE activity, alongside with receptors to AngII, which allowed chemotaxis and proliferation. Remarkably, the use of RASB did not completely reverse the effects of AngII, which suggested the presence of an indirect pathway, or a different receptor to AngII [169]. Consequently, the development of a selective AT2 receptor agonist, compound 21, may hold promise to mitigate immune-mediated endothelial inflammation [180] and subsequent allograft vasculature damage.

While overly simplified, inflammation may be associated with an increased risk of transplant rejection, and, hence, it can constitute a legitimate therapeutic target in transplant recipients [181]; however, the lack of selective RAS inhibitor, capable of distinguishing between local target allograft vasculature and systemic use, precludes, for now, relevant therapeutic options, other than usual RASB, for which studies may only yield overall net effects of systemically blocking RAS.

### 4.2. RAS in Heart Transplantation

The use of RASB in heart transplant recipients has been associated with a decrease in the development of cardiac allograft vasculopathy; results, however, were conflicting in their magnitudes. A randomized controlled trial in 96 patients, comparing ramipril to a placebo, did not show actual coronary plaque volume decrease with ramipril, yet, microvascular function was improved, as was the amount of endothelial progenitor cells [182]. Interestingly, assessing the levels of RAS components in these patients showed that patients treated using RASB sometimes showed higher AngII levels than before being treated with RASB [183], a phenomenon called ACEIs-escape [184], contributing to chronic activation of RAS. The main issue lies in the fact that AngII may be independent from ACE in some cases and several alternate pathways have been discussed as plausible substrates to produce AngII, specifically in human cardiac tissues. Chymase, a serine protease, was described as such [184].

ACEIs inhibits the transformation of AngI to AngII, but as a result, increases the production of Ang 1–7 (through Ang 1–9 with ACE2 then NEP, or straight through NEP), which, in turn, may be associated with anti-inflammatory and anti-fibrotic effects. As ACE levels depend on localization, a distinction must be made between local tissue levels and systemic plasma levels of circulating peptides. In a biological study, Kovaric et al. sampled RAS components in plasma versus cardiac tissue in 25 heart transplant recipients, 15 of whom were treated using ACEIs. They showed that while systemic AngII formation depended on ACE, local formation of AngII in cardiac graft depended on chymase activity [185]. These findings emphasized the previously described importance of ACE-independent AngII formation pathways in the myocardium [186]. Of note, this study noted that Ang 1–7 plasma levels increased in patients treated using ACEIs, either through accumulation of AngI or through the increase of cardiac NEP activity.

Hence, in the near future, use of NEP inhibitors, such as sacubitril, may yield beneficial results in heart transplant recipients, as previously demonstrated in reduced rejection heart failure patients [187].

### 4.3. RAS in Kidney Transplantation

Kidney transplantation presents the particularity that RAS is a direct function of the allograft. Indeed, preserving local vasculature from injury and inflammation holds the double purpose of preventing RAS activation through blood-pressure control and graft preservation.

As described above for heart transplantation, AngII formation may be independent of ACE, with a progressive switch after kidney transplantation. In their study, Kovaric et al. hypothesized that, in the first months, AngII occurs in the graft through moderate ACE activity, while Ang 1–7 formation occurs through NEP and ACE-mediated AngII (from AngI). Several years later, chymase activity becomes prominent and takes over the previously ACE-mediated AngII formation, alongside the persisting NEP-mediated Ang 1–7 formation [188].

The effects of RASB treatments in kidney transplant recipients are debated. Similar to what has been observed in heart transplantation, some benefits have been hypothesized in various observational cohorts [189,190,191]. However, a randomized controlled trial which included 213 patients with proteinuria, after kidney transplantation, and compared ramipril to a placebo, did not yield significant benefits of RAS blockade [192]. Its lack of power, relative to a number of inclusions lower than that which was required to achieve the proper beta, was invoked, despite a longer-than-planned follow-up. Similarly, the number of patients which were required to demonstrate similar improvements in clinical outcomes, i.e., patients with diabetes mellitus and chronic kidney disease, required thousands of patients, far above the 213 included in the study. Hence, it is not clear whether RASB may improve transplant survival after kidney transplantation and investigations are still ongoing.

## 5. Conclusions

The renin–angiotensin system is usually associated with blood pressure homeostasis, and, yet, its impact on immunomodulation, which encompass various entities, such as cancer, septic shock, and transplantation, should warrant a physician’s awareness on potential therapeutic targets using readily available drugs.

## Figures and Tables

**Table 1:** Summary of meta-analyses of RABs use and overall risk of cancer occurrence. Abbreviations: ACEIs: angiotensin-converting enzyme inhibitors, ARBs: angiotensin-2 receptor blockers, CI: confidence interval, HR: hazard ratio, OR: odds ratio, RABs: renin-angiotensin system blockers, RR: risk ratio.

Authors	Date	Number of Studies	Type of Studies	Number of Patients	Results
Sipahi et al. [55]	2010	9	Randomized controlled trials. Condition studied was cardiovascular (hypertension, heart failure, myocardial infarction, etc.)	- 61,590 patients from five trials had data on new cancer occurrence - 68,402 patients from five trials had data on common types of solid organ cancers - 93,515 patients from eight trials had data on cancer deaths	- Significantly increased risk of new cancer occurrence: (7.2% vs. 6.0%, RR 1.08, 95% CI 1.01–1.15; *p* = 0.016). - No statistically significant difference in cancer deaths (1.8% vs. 1.6%, RR 1.07, 0.97–1.18; *p* = 0.183).
Yoon et al. [57]	2011	28	Observational: - Cohort *n* = 12 (Two of the cohort studies shared a study population were used only in the subgroup analyses) - Nested case–control *n* = 6 - Conventional case–control *n* = 10	- 3,611,694 patients included in the cohort studies, among them 26,912 were RABs users. - All but 2 of the 16 case–control studies reported the number of cases (*n* = 27,987) and controls (*n* = 119,879). 2043 (7.3%) among the cases and 9470 (7.9%) among the controls were RABs users.	- No significant association between the use of RABs and the overall risk of cancer: RR 0.96, 95% CI 0.90–1.03. - Decreased risk of cancer when authors restricted the analyses to cohort and nested case–control studies: RR 0.90, 95% CI 0.83–0.97; or to studies with long-term follow-up of more than five years: RR 0.89, 95% CI 0.83–0.96.
Shen et al. [56]	2016	31	- 14 randomized trials - 17 observational studies	- 3,957,725 participants, among them 35,329 were ARB/ACEIs users.	- Decrease incidence of cancer in RABs users in the observational studies (RR 0.82, 95% CI 0.73–0.93) but not in the randomized controlled trials (RR 1.00, 95% CI 0.92–1.08). - Significant reduction of mortality with ARB/ACEIs in the observational studies (RR 0.71, 95% CI 0.55–0.93) but not in the randomized controlled trials (RR 0.99, 95% CI 0.89–1.09).

**Table 2:** Renin–angiotensin receptor blockers and specific organ cancer risks. Abbreviations: ACEIs: angiotensin-converting enzyme inhibitors, ARBs: angiotensin-2 receptor 1 blockers, BCC: basal cell carcinoma, CI: confidence interval, CRC: colorectal cancer, HR: Hazard ratio, OR: odds ratio, RR: risk ratio, SCC: squamous cell carcinoma, UK: United Kingdom.

Authors	Date	Type of Trial	Type of Cancer	Number of Patients	Results for Length of Treatment	Results for Risk of Cancer
Dai et al. [58]	2015	Meta-analysis of six observational studies	CRC	113,048 individuals	-	Decreased risk of CRC in RABs users compared to non-users: RR 0.94 95% CI 0.89–0.98, *p* = 0.006.
Cheung et al. [59]	2020	Retrospective cohort study	CRC	187,897 patients, 30,856 (16.4%) were RABs users	-	Lower risk of CRC that developed <3 years after index colonoscopy (HR, 0.78 (95% CI, 0.64–0.96)), but not CRC that developed >3 years (adjusted hazard ratio, 1.18 (95% CI, 0.88–1.57)).
Christian et al. [60]	2008	Prospective randomized study	Keratinocyte	1051 individuals	-	Significantly reduction of risks of BCC (IRR = 0.61, 95% CI = 0.50 to 0.76) and SCC (IRR = 0.67, 95% CI = 0.52 to 0.87).
Mao et al. [61]	2016	Meta-analysis of nine cohort studies	Prostate	20,267 patients	-	Using RABs inhibitors reduced the risk of prostate cancer: RR 0.92, 95% CI 0.87–0.98.
Hagberg et al. [62]	2016	Nested case-control study	Liver	490 cases with hypertension and liver cancer, 1909 controls	No significant difference in risk by duration of use.	No association between use of RABs and the risk of liver cancer: OR 1.13 95% CI 0.79–1.60.
Xie et al. [65]	2020	Meta-analysis of 31 trials: 18 case-control studies and 13 cohort studies.	Kidney and bladder cancer	3,352,264 patients	-	Significant association between the risk of kidney cancer and ACEIs use RR 1.24, 95% CI 1.04–1.48, as ARBs use: RR 1.29, 95% CI:1.22–1.37. Increase risk of bladder cancer with ARBs use: RR 1.07, 95% CI 1.03–1.11.
Qian et al. [63]	2017	Meta-analysis of eight studies	Breast	1,994,880 individuals	-	No association between ARBs use and the risk of breast cancer: OR 0.93; 95% CI: 0.81–1.06.
Ni et al. [64]	2017	Meta-analysis of 21 studies: 9 prospective cohort studies and 12 case-control studies	Breast	3,116,266 participants	Reduced breast cancer risk for RABs use ≥10 years: RR 0.80, 95% CI: 0.67–0.95.	ACEIs/ARBs use was not significantly associated with breast cancer risk: RR 0.99 95% CI: 0.93–1.05.
Hicks et al. [68]	2018	Population-based cohort study (UK)	Lung	992,061 patients	HR gradually increased with longer durations of use: - after five years of use: HR 1.22, 1.06 to 1.40. - after more than 10 years of use: HR 1.31, 1.08 to 1.59.	Increased risk of lung cancer with ACEISs use (incidence rate 1.6 v 1.2 per 1000 person years; HR 1.14, 95% CI 1.01 to 1.29), compared with use of ARBs.
Lin et al. [69]	2020	Population-based, propensity score-matched cohort study	Lung	22,384 ACEIs and 22,384 ARBs users	Significantly higher risk in patients who received more than 540 defined daily doses of ACEIs per year: HR 1.80; 95% CI 1.43–2.27	Increased risk of lung cancer with ACEIs use HR = 1.36 95% CI = 1.11–1.67, compared with use of ARBs.

**Table 3:** Summary of RAB and cancer survival studies. Abbreviations: ACEIs: angiotensin-converting enzyme inhibitors, ARBs: angiotensin-2 receptor blockers, CI: confidence interval, CRC: colorectal cancer, DFS: disease free survival; HR: hazard ratio, NSCLC: non-small cell lung cancer, OR: odds ratio, RABs: renin-angiotensin-aldosterone system blockers, RFS: recurrence free survival, RR: risk ratio.

Authors	Date	Type of Trial	Type of Cancer	Number of Patients	Results
Song et al. [79]	2016	Meta-analysis from 11 studies: 9 retrospective and 2 prospective hospital-based cohort	Overall: urinary tract, prostate, breast, CRC, hepatocellular, pancreatic, NSCLC	- 4964 patients - 750 RABs users and 4214 nonusers	- Significant improvement in use of RABs on DFS: HR 0.60; 95% CI 0.41–0.87; *p* = 0.007, and overall survival: HR 0.75; 95% CI 0.57–0.99; *p* = 0.04. - DFS improvement was applied to both low stage (I/II) HR 0.56; 95% CI 0.32–0.96; *p* = 0.04; and high stage (III/IV): HR 0.59; 95% CI 0.37–0.94; *p* = 0.03.
Li et al. [80]	2017	Meta-analysis from 7 retrospective trials	Advanced cancers: NSCLC, pancreatic, gastric cancer, breast, renal	- 2436 patients - 378 in the chemotherapeutic agents RABs groups	- Significant reduction in overall mortality in favor of chemotherapeutic agents in combination with RABs agents: HR 0.80, 95% CI: 0.69–0.92. - Significant decrease in the risk of disease progression in favor of chemotherapeutic agents in combination with RABs regimens: HR 0.79, 95% CI: 0.66–0.94.
Morris et al. [81]	2015	Retrospective study in 2 centers	CRC treated with neoadjuvant radiation	- 301 patients - 74 taking RABs	- Multivariate analyses identified RABS use as a strong predictor of pathologic complete response: OR 4.02; 95% CI 2.06–7.82; *p* < 0.001.
Ozawa et al. [82]	2019	Retrospective, monocentric	CRC stage I-III	- 461 patients	- The Kaplan-Meier curves showed a trend toward improved RFS in RABs users (*p* = 0.063). - In subgroup analyses, RFS was significantly better in RABs users in the patients with left-sided CRC (*p* = 0.030) and those with stage I CRC (*p* = 0.009).
Zhou et al. [83]	2020	Meta-analysis from 13:12 cohort and 1 randomised controlled study	Digestive system malignancies: CRC, pancreatic, liver, gastric	-	- Use of RABs resulted in a significant improvement in overall survival: HR 0.79; 95% CI 0.70–0.89; *p* < 0.0001. - Two studies evaluated the dose–response and showed that higher doses of RABS lead to better survival: 1–364 defined daily doses: OR 0.89, 95% CI 0.78–1.01, *p* = 0.076; ≥365 defined daily doses: OR 0.54, 95% CI: 0.24–1.24, *p* = 0.148.
Santala et al. [84]	2020	Nationwide cohort study (Finland)	Breast	- 73,170 women	- In prediagnostic use, only ARBs were associated with decreased risk of breast cancer death: HR: 0.76, 95% CI: 0.69–0.82. In postdiagnostic use, there were a dose dependent increase of breast cancer survival for both ARBs: HR 0.69, 95% CI: 0.63–0.75; and ACEIs: HR 0.92, 95% CI 0.86–0.98.
Menter et al. [90]	2017	Retrospective cohort study	Advanced NSCLC	- 1813 patients - 351 received RABs	- In propensity score matched cohort analysis, concomitant RABs use increase survival for patients receiving carboplatin and paclitaxel: HR 0.73, 95% CI 0.61–0.88, and for patients receiving carboplatin and paclitaxel with bevacizumab: HR 0.79, 95% CI 0.51–1.21.
Wilop et al. [91]	2009	Retrospective cohort study	Advanced NSCLC treated by first-line platinum-based chemotherapy	- 287 patients - 52 (18.1%) received RABs	- Increase of median survival in RABs users: 11.7 vs. 8.6 months, HR 0.56, *p* = 0.03.
Medjebar et al. [92]	2020	Retrospective cohort study	NSCLC treated with PD-1/PD-L1 immune checkpoint blockers.	- 178 patients - 22 (13.1%) received RABs	- Shorter median progression-free survival in ACEIs users compared to the control group: 1.97 vs. 2.56 months: HR 1.8, CI 95% 1.1–2.8, *p* = 0.01. - ACE inhibitors group had less M1 macrophages, activated mast cells, NK cells and memory activated T cells, thus suggesting an immunosuppressed state.
Santala et al. [89]	2019	Nationwide cohort study (Finland)	Bladder cancer and upper tract urothelial carcinomas	- 15,145 patients - 8393 using ACEIs/ARBs.	- ARBs use before diagnosis w risk of bladder cancer death: HR = 0.80, CI 0.70–0.92. The association was dose-dependent. No association with ACEIs use. - Post-diagnostic use of ARBs was similarly associated with better survival: HR 0.81, CI = 0.71–0.92.
Asgharzadeh et al. [86]	2020	Meta-analysis from nine studies	Renal cancer	-	- Significantly lower mortality with RABs use: HR 0.723, 95% CI 0.568–0.921, *p* = 0.009. - Higher mortality in ACEIs users: HR 1.352, 95% CI 0.917–1.991, *p* = 0.128. - Decreased of mortality in ARBs users: HR 0.818, 95% CI 0.691–0.969, *p* = 0.02.
Alashkham et al. [85]	2016	Retrospective cohort study	Prostate cancer after radical radiotherapy with adjuvant/neoadjuvant hormone treatment	558 patients	- In a propensity score analysis, there were a significant reduction of incidence of biochemical recurrence in hypertensive men taking ACEIs/ARBs than in non-hypertensive men not taking RABs: RR 0.74; 95% CI 0.64–0.86; *p* < 0.001, or in hypertensive men taking other anti-hypertensive drugs: RR 0.78; 95% CI 0.67–0.91; *p* < 0.001.
Yoshida et al. [88]	2017	Retrospective cohort study	Bladder cancer after radical cystectomy	269 patients	- Significant increase in 5-year cancer-specific survival rates in patients who receive RABs (79.0 and 66.4%, *p* = 0.011) - In the multivariable analyses, RABs use was an independent prognostic factor for cancer-specific survival: HR 0.47, *p* = 0.036; and for overall survival (HR 0.36, *p* = 0.022).
Blute et al. [87]	2015	Retrospective cohort study	Bladder cancer after initial transurethral resection	340 patients	- Significantly reduction of tumor recurrence in multivariate analysis with RABs therapy: HR 0.61, 95% CI 0.45–0.84, *p* = 0.005. - After exclusion of non-invasive bladder cancer, adjunction of RABs to bacillus Calmette-Guérin therapy increase the recurrence-free-survival: HR 0.45, 95% CI 0.21–0.98, *p* = 0.04.

**Table 4:** Summary of studies about RAB use to prevent cardiotoxicity in breast cancer. Abbreviations: CI: confidence interval, HR: hazard ratio, LEVF: left ejection ventricular fraction, OR: odds ratio, RABs: renin-angiotensin-aldosterone system blockers.

Authors	Date	Type of Trial	Anti-Cancer Therapy/Intervention	Number of Patients	Results
Gulati et al. [75]	2016	Randomized, placebo-controlled, double-blind trial	Anthracycline ± trastuzumab and radiation Cansartan, metoprolol, placebo	130 women	- Significant reduction of LEVF decrease (evaluated by MRI), with candesartan: the decline of LVEF was 2.6% (95% CI 1.5–3.8) in the placebo group and 0.8 (95% CI 0.4–1.9) in the candesartan group; *p* = 0.026. No effect of metoprolol on the overall decline in LVEF was observed.
Pituskin et al. [73]	2016	Randomized, placebo-controlled trial	Trastuzumab Perindopril, bisoprolol, placebo (1:1:1)	94 women	- Trastuzumab-mediated decline in LVEF was attenuated in bisoprolol-treated patients (−1 ± 5%) relative to the perindopril (−3 ± 4%) and placebo (−5 ± 5%) groups (*p* = 0.001) - Perindopril and bisoprolol use were independent predictors of maintained LVEF on multivariable analysis. - Ventricular remodeling, the primary outcome, was not prevented by these pharmacotherapies.
Guglin et al. [71]	2019	Randomized, double-blind, multicenter, placebo-controlled trial	- Trastuzumab, Anthracyclines - lisinopril, carvedilol, or placebo (1:1:1)	468 women, age 51 ± 10.7 years	- Cardiotoxicity-free survival was longer on lisinopril: HR 0.53; 95% CI 0.30–0.94; *p* = 0.015; than on placebo. - Reduction of the event rate for patients receiving anthracyclines in the lisinopril (37%) than in the placebo group (47%).
Wittayanukorn et al. [72]	2018	Retrospective cohort study	Trastuzumab and anthracycline	6542 women (66 years-old and above): 508 (7.7%) exposed to RABs	- RABs use reduced cardiotoxicity: HR 0.77, 95% CI 0.62–0.95; and all-cause mortality: HR 0.79, 95% CI 0.70–0.90, compared with the nonexposed group. - Dose-dependent interaction: starting of RABs therapy ≤6 months after the initiation of trastuzumab/anthracyclines and having exposed duration ≥6 months were also associated with decreased risk of cardiotoxicity and all-cause mortality.
Moey et al. [74]	2019	Retrospective cohort study	Trastuzumab	127 women	- 13 (11%) developed cardiotoxicity resulting in discontinuation of trastuzumab. - Patients who received RABs were less likely to developed cardiotoxicity, defined by a reduction of more than 15% of LEVF: OR 0.24, 95% CI 0.05–1.11, *p* = 0.06.

## Abbreviations

ACE	angiotensin-converting enzyme
ACE2	angiotensin-converting enzyme type 2
ACEIs	angiotensin-converting enzyme inhibitors
AngI	angiotensin I
AngII	angiotensin II
Ang 1–7	angiotensin 1–7
Ang 1–9	angiotensin 1–9
ARBs	angiotensin-2 receptor 1 blockers
AT1R	angiotensin II receptor type 1
AT2R	angiotensin II receptor type 2
NEP	neprilysin
NK	natural killer
RAS	renin–angiotensin system
ROS	reactive oxygen species
VEGF	vascular endothelial growth factor
